# Control of Aflatoxin Production of *Aspergillus flavus* and *Aspergillus parasiticus* Using RNA Silencing Technology by Targeting *aflD* (nor-1) Gene

**DOI:** 10.3390/toxins3060647

**Published:** 2011-06-17

**Authors:** Ahmed M. Abdel-Hadi, Daniel P. Caley, David R. F. Carter, Naresh Magan

**Affiliations:** 1 Applied Mycology Group, Cranfield Health, Vincent Building, Cranfield University, Bedford MK43 0AL, UK; Email: ahmed_alhadi2000@yahoo.com; 2 Current address—School of Life Sciences, Oxford Brookes University, Oxford, OX3 0BP, UK; Email: d.cayley@brookes.ac.uk (D.P.C.); d.carter@brookes.ac.uk (D.R.F.C.)

**Keywords:** siRNA, *aflD* (nor-1) gene, *aflR* gene, aflatoxin, real-time PCR

## Abstract

*Aspergillus ﬂavus* and *Aspergillus parasiticus* are important pathogens of cotton, corn, peanuts and other oil-seed crops, producing toxins both in the field and during storage. We have designed three siRNA sequences (Nor-Ia, Nor-Ib, Nor-Ic) to target the mRNA sequence of the *aflD* gene to examine the potential for using RNA silencing technology to control aflatoxin production. Thus, the effect of siRNAs targeting of two key genes in the aflatoxin biosynthetic pathway, *aflD *(structural) and *aflR* (regulatory gene) and on aflatoxin B_1 _(AFB_1_), and aflatoxin G_1_ (AFG_1_) production was examined. The study showed that Nor-Ib gave a significant decrease in *aflD* mRNA, *aflR* mRNA abundance, and AFB_1_ production (98, 97 and 97% when compared to the controls) in *A. flavus* NRRL3357, respectively. Reduction in *aflD *and *aflR* mRNA abundance and AFB_1 _production increased with concentration of siRNA tested. There was a significant inhibition in *aflD* and AFB_1_ production by *A. flavus* EGP9 and AFG_1 _production by *A. parasiticus *NRRL 13005. However, there was no significant decrease in AFG_1_ production by *A. parasiticus* SSWT 2999. Changes in AFB_1_ production in relation to mRNA levels of *aflD* showed a good correlation (*R* = 0.88; *P* = 0.00001); changes in *aflR *mRNA level in relation to mRNA level of *aflD* also showed good correlation (*R* = 0.82; *P* = 0.0001). The correlations between changes in a*flR *and *aflD* gene expression suggests a strong relationship between these structural and regulatory genes, and that *aflD* could be used as a target gene to develop efficient means for aflatoxin control using RNA silencing technology.

## 1. Introduction

Aﬂatoxins are secondary metabolites produced by *Aspergillus ﬂavus* and *A. parasiticus *that occur in nuts and cereal crops. These compounds have a high acute toxicity, as well as immunosuppressive, mutagenic, teratogenic, and carcinogenic activities and are classiﬁed as group 1 carcinogens by the International Agency for Research on Cancer [1].

Controlling aflatoxin production is of critical importance. Mainly traditional control methods including cultural practices such as pesticides and the development of resistant cultivars, pest resistance have been used. However, these have not always been successful in maize and in groundnuts. There is thus interest in exploring alternative means to control or reduce aflatoxin production. 

RNA interference technology (RNAi) has received much attention in biology. The reason for this enthusiasm is that RNAi can rapidly ablate specific messenger RNA (mRNA) species by inducing their degradation via cellular protein machinery collectively named the RNA-induced silencing complex [2]. Short-interfering double-stranded RNA (siRNA) is synthesized and introduced through common transfection methods into cells, where they serve to guide the RNA degradation machinery to the select target gene. RNAi is an effective tool to investigate gene function, and may also be a useful tool to quench the expression of undesirable gene products. 

RNA silencing in filamentous fungi has been carried out using plasmid constructs expressing a hairpin dsRNA structure controlled by an inducible or constitutive promoter [3,4,5,6]. Liu *et al.* [7] demonstrated silencing of the cryptococcal *CAP59* and *ADE2* genes by double-stranded RNA homologous to these genes in the basidiomycetous yeast *Cryptococcus neoformans*. 

Application of siRNA-mediated RNAi has also been reported in cultured cells from fungi. Katri and Rajam [8] reported that ornithine decarboxylase (ODC) was specifically silenced by treating germinating spores with synthetic 23 nt siRNA in *Aspergillus nidulans*. Doubled-strand of RNA (dsRNA) was also delivered directly into protoplasts of *Phytophthora infestans*, which belongs to the fungus-like Oomycetes [9]. 

In *A. flavus* and *A. parasiticus* the expression of the *aflD* (nor-1), a gene encoding an enzyme that catalyzes the conversion of the first stable aflatoxin biosynthesis intermediate, norsolorinic acid, to averantin [10,11] is a key structural gene in the biosynthetic pathway. Furthermore, *aflR* is a pathway regulatory gene coding for proteins shown to be involved in transcriptional activation of most of the structural genes [12]. Recent studies have shown that there may be a relationship between the ratio of *aflR* and *aflS* (the associated regulatory gene) genes which is influenced by environmental factors [13]. Recently, studies by Abdel-Hadi *et al.* [14,15] showed the potential use of *aflD* transcription as a good marker to discriminate between aflatoxigenic and non-aflatoxigenic strains contaminating peanuts while* aflR* failed to differentiate between these strains. They showed that the expression patterns of *aflD* were related to changing water activity in stored peanuts. In peanuts, *aflR* was found not to change in the same consistent way with water availability in peanuts. Thus, the expression pattern of this structural gene was selected as a target gene for silencing.

The objective of this study was to determine the potential of siRNA for silencing the target gene (*aflD*) and phenotypic aflatoxin control in strains of *A. flavus* and *A. parasiticus*. 

## 2. Materials and Methods

### 2.1. Fungal Strain and Growth Conditions

In this study, four aflatoxigenic strains (*Aspergillus flavus* NRRL3357, *Aspergillus flavus *EGP9, *Aspergillus parasiticus *NRRL 13005 and *Aspergillus parasiticus* SSWT 2999) have been used. The strains were sub-cultured on Malt Extract Agar (20 g malt extract, 2 g peptone, 15 g agar per liter) for 7 days at 25 °C in the dark.

### 2.2. Preparation of Protoplast

Protoplasts were prepared from actively growing mycelium; a spore suspension of the strains sub-cultured in 200 mL of Yeast Extract Sucrose (YES) broth (20 g yeast extract, 150 g sucrose per liter), then incubated for 24 h on a shaker at 200 rpm in the dark at 25 °C. The mycelium was harvested by filtration through Miracloth. One gram of mycelia was transferred into 20 mL of filter sterilized enzyme solution (per 20 mL: 17 mL of H_2_O, 2 mL of 0.2 M NaPO_4_ (pH 5.8), 0.4 mL of 1.0 M CaCl_2_, 1.4 g of NaCl, 0.2 mL of β-glucuronidase (105 U/mL; Sigma), 200 mg of lysing enzyme (Sigma), and 50 mg of driselase (Sigma). Mycelia were incubated at 30 °C with shaking (80 rpm) for 3 h. Protoplasts were separated from intact mycelia by passage through Miracloth into a sterile 50 mL tube, and 20 mL of sterile STC buffer (1.2 M sorbitol, 10 mM CaCl_2_, 10 mM Tris-HCl (pH 7.5)) was added. Protoplasts were pelleted by low-speed centrifugation (1000 rpm) at room temperature for 5 min. The supernatant was carefully removed, and the protoplasts were washed once more in 20 mL of STC and pelleted by centrifugation as described previously. The protoplast pellet was resuspended in 1.0 mL of STC buffer, and the protoplasts were counted on a haemocytometer and diluted to 1 × 10^5^/mL [16]. 

*siRNA design: *Three siRNA sequences were designed by Ambion (Applied Biosystem) to target the mRNA sequence of the *aflD* gene of *A. flavus* (accession number EF565463**) **and purchased from the same company. These siRNA were named as Nor-Ia, Nor-Ib and Nor-Ic (Table 1). Annealing of RNA oligonucleotides and purification by HPLC were performed by the company. An siRNA (control-siRNA) with no sequence homology to any *A flavus* genome sequence database was also purchased from Ambion. 

**Table 1:** Details of siRNA sequences used in this study.

siRNA Name	siRNA Sequence
Nor-Ia	Sense strand: CAUGUAUGCUCCCGUCCUAUU
	Antisense strand : UAGGACGGGAGCAUACAUGUU
Nor-Ib	Sense strand: GCAACAGGCCAAGUUUGUCUU
	Antisense strand : GACAAACUUGGCCUGUUGCUU
Nor-Ic	Sense strand: CAGGCCAAGUUUGUCUUGAUU
	Antisense strand : UCAAGACAAACUUGGCCUGUU

### 2.3. Delivery of siRNA to Protoplast

All siRNAs were resuspended in water free of RNases at a final concentration of 25 nM and tested on *A. flavus* NRRL3357. In a sterile 1.5 mL micro centrifuge tubes, 10 µL of each siRNA was mixed with an equal volume of Lipofectin reagent (Invitrogen Life Technologies, UK) and allowed to stand for 15 min at 20 °C. 20 µL of protoplasts (1 × 10^3^) were added and mixed gently. The tubes were incubated at 20 °C for 24 h to allow transfection to proceed [9]. Then the mixture was inoculated in 10 mL of YES medium with 1.2 M of sorbitol for 5 days at 25 °C in the dark. Different dilutions of Nor-Ib (5, 10, 15, 20, 25 nM) were tested on *A. flavus* NRRL3357. Twenty five nM of Nor-Ib was tested on *Aspergillus flavus *EGP9, *Aspergillus parasiticus *NRRL 13005 and *Aspergillus parasiticus* SSWT 2999. All experiments were carried out using three biological replicates.

### 2.4. Aflatoxin Extraction and HPLC Analysis

Five mL of filtrate was extracted with chloroform, and then the extract was evaporated. The residue was derivatized using TFA (Triflouroacetic acid) as described by the AOAC [17]. Sample extracts were analyzed using an Agilent 1200 series HPLC (Agilent, Berkshire, UK) using a 470 fluorescence detector (FLD, G1321A, Agilent) (λ_exc _360 nm; λ_em _440 nm) and a C_18_ column (Phenomenex Luna ODS2 150 × 4.6 mm, 5 µm; Maccesfield, UK). The analysis was performed using a mobile phase of methanol: water: acetonitrile (30:60:10) at a flow rate of 1 mL/min and a run time of 25 min.

### 2.5. Isolation of RNA from the Samples and RT-PCR

Total RNA was extracted from mycelium using the RNeasy and Plant Mini Kit (Qiagen GmbH, Hilden, Germany). A 0.5–1.0 g sub-sample of the mycelia was ground in a mortar with a pestle in liquid nitrogen. Approximately 250 mg of the mycelial powder was then used for isolation of total RNA. RNA extraction from the ground mycelia was accomplished with the RNeasy and Plant Mini Kit (Qiagen GmbH, Hilden, Germany) according to the instructions provided by the manufacturer. Then RNA was treated with DNase I (RNase free DNase I, Amplification Grade, Sigma) to digest residual DNA in the samples.

**Figure 1:**
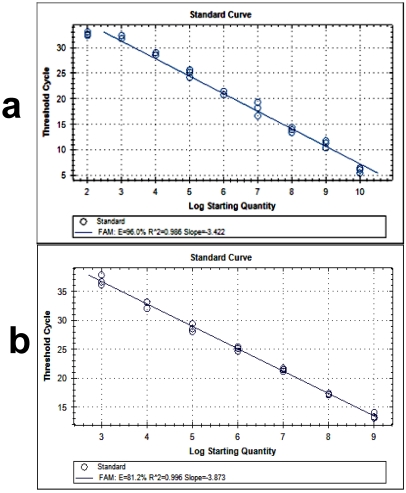
Standard curves from real-time PCR by plotting the threshold cycle (Ct) *vs. *log_10_ initial copy numbers of *aflD* gene (**a**) and *aflR* gene (**b**) amplified with the primer of labeled with FAM. Where E: The efficiency of PCR, R^2^ value: correlation coefficient.

*TaqMan probes and primer design: *Real Time RT-PCR was used to amplify the *aflD *gene (target gene) and *aflR *gene (regulatory gene). The two primers and an internal fluorescence labelled probe used in the reaction were nortaq-1 5'-GTCCAAGCAACAGGCCAAGT-3'; nortaq-2 5'-TCG TGCATGTTGGTGATGGT-3'; norprobe 6FAM TGTCTTGATCGCGCCCG- BHQ2 [18]; AflRtaq-1 5'-TCGTCCTTATCGTTCTCAAGG-3'; AflRtaq-2 5'-ACTGTTGCTACAGCTGCCACT-3', AflRprobe 6FAM AGCAGGCACCCAGTGTACCTCAAC-BHQ2. To create a standard curve, a larger PCR fragment of the *aflD* (nor-1) gene was generated with the primer nor1 and nor2 [19] (Figure 1a). Different dilutions were prepared from a stock solution by a factor of 10 and the aliquots of the dilutions were used in standard reactions during each setup of the real-time PCR reaction. The concentration of this standard PCR product was determined in a spectrophotometer (WPA light wave Cambridge, UK) and the number of copies was calculated. The concentration of unknown samples was calculated by the CFX96 system (Bio Rad, Hercules, CA) according to the generated standard curve. To create a standard curve for *aflR*, a larger PCR fragment of the *aflR* gene was used. To create the standard curve, a larger PCR fragment of the *aflR *with the following primers AflR1, 5'-CGAGTTGTGCCAGTTCAAAA-3'; AflR2, 5'-AATCCTCGCCCACCATACTA-3' was used (Figure 1b).

*Real-time PCR conditions: *Amplification was performed using a total reaction volume of 25 µL in a MicroAmp optical 96-well reaction plate (Applied Biosystems). For each reaction 12.5 µL of TaqMan Universal Master Mix (Applied Biosystems), 2.5 µL cDNA, 3 µL of primer and probe mix (0.5 nM primer and 0.2 nM probe), and 7 µL of free RNases water. Real Time reactions were performed using the Bio Rad CFX96 platform (Bio Rad, Hercules, CA) with the following conditions: an initial step at 95 °C for 10 min, and all 40 cycles at 95 °C for 15 s, 55 °C for 20 s and 72 °C for 30 s.

*Statistical analysis: *All experiments were carried out with 3–4 replicates and repeated twice with similar results. Statistical tests were performed using Statistica version 8 (StatSoft, Inc, 1984–2007) for one-way ANOVA and LSD Fisher was determined at the 95% confidence limits. 

## 3. Results

### 3.1. Treatment of *Aspergillus flavus* NRRL with siRNA

**Figure 2:**
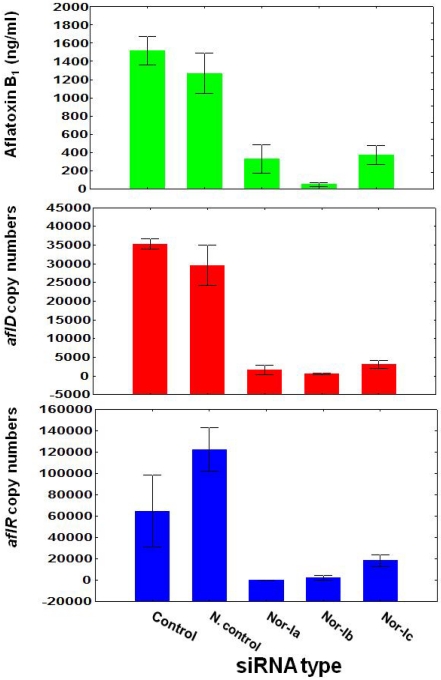
Effect of siRNA for silencing *aflD* target gene on aflatoxin B_1_ production, gene expression of *aflD* and *aflR* by using real-time PCR of *Aspergillus flavus *NRRL. Vertical bar indicates standard error, control (untreated with siRNA), and N. Control (treated with unrelated siRNA as a negative control).

Quantification of the *aflD* based on the absolute quantification of copy number by using the calibration curve provided accurate, sensitive and highly reproducible data [20]. Figure 2 shows the changes in *aflD* and *aflR* mRNA expression and AFB_1_ production by *A. flavus *NRRL3357 after treatment with control-siRNA or three siRNAs (Nor-Ia, Nor-Ib, Nor-Ic) specific to the *aflD* target gene. Treatment with the control-siRNA had no significant effect on AFB_1_ production or *aflD*/*aflR* mRNA copy numbers. There was a significant decrease (95, 98, and 91% of the control level) in *aflD* mRNA abundance after treating with Nor-Ia, Nor-Ib and Nor-Ic siRNAs respectively, as assessed by real-time PCR. The lack of any effect using the control-siRNA and the knockdown seen with all three *aflD* siRNAs suggested that the results were not caused by transfection conditions or due to off-target effects. 

Interestingly, a decrease (99% (Nor-Ia treatment), 97% (nor-Ib treatment), and 72% (Nor-Ic treatment) of the control level in *aflR* mRNA abundance was also observed following knockdown of *aflD*. Subsequently, a decrease of AFB_1_ production as a result of a decrease of *aflD* and *aflR *gene expression ((79% (Nor-1a treated), 97% (Nor-Ib treated), and 76% (Nor-Ic treated)) of the control level was obtained. Statistical analysis of the effect of siRNA treatment on *aflD *gene expression, *aflR* gene expression and AFB_1_ production were highly significant (Table 2a). There was a good correlation between siRNA effects on *aflD *and *aflR *expression (*R* = 0.82, *P* = 0.0001); *aflD* and AFB_1_ (*R* = 0.88, *P* = 0.00001); and *aflR *correlated significantly with AFB_1_ (*R* = 0.66, *P *= 0.0074) (Table 3). 

**Table 2:** (**a**) Analysis of Variance of the effect of siRNA silencing of the *aflD* target gene on AFB_1_ production, expression of *aflD* gene and *aflR* gene, and (**b**) effect of different concentrations of siRNA (Nor-Ib) on log AFB_1_ *production*, log quantification of *aflD* gene and *aflR* gene. Key: DF: Degrees of freedom, MS: mean square; P: Probability, F: F value.

	DF	MS	F	P
(a)				
Factor				
*aflD* copy numbers	4	8.58 × 10^8^	42.47	0.000003
AFB_1_	4	1.24 × 10^8^	18.74	0.0001
*aflR* copy numbers	4	8.16 × 10^9^	8.63	0.0027
(b)				
Factor				
log *aflD *	5	1.87	10.95	0.0003
log AFB_1_	5	0.41	199.13	0.00000
log *aflR*	5	2.4659	6.05	0.005

**Table 3:** Statistical correlations between *aflD* gene, *aflR *gene and AFB_1_ production of *A. flavus* NRRL3357 treated with siRNA (Nor-Ib). Key: R: correlation coefficient, P: Probability, F: F value.

Correlations	R Value	F	P
*aflD andaflR*	0.82	28.41	0.0001
*aflD* and AFB_1_	0.88	47.26	0.00001
*aflR* and AFB_1_	0.66	10.039	0.0074
log *aflD and* siRNA conc.	0.86	46.31	0.000
log AFB_1 _and siRNA conc.	0.91	77.75	0.000
log *aflR* and siRNA conc.	0.45	4.07	0.06

### 3.2. Effect of siRNA Concentrations on *A. flavus* NRRL3357

**Figure 3:**
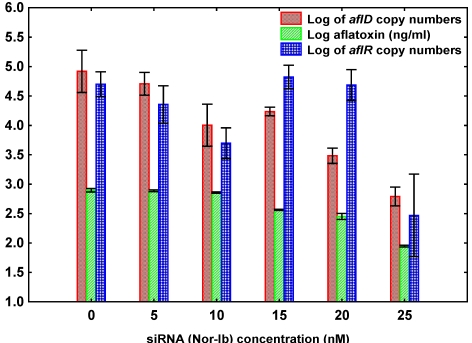
Effect of different concentrations of siRNA (Nor-Ib) on Aflatoxin B_1_ production, gene expression of *aflD* and *aflR* by using real-time PCR of *Aspergillus flavus *NRRL3357. Vertical bar indicate standard errors of the mean.

Figure 3 compares the effect of different concentrations of siRNA (Nor-Ib) on quantification of *aflD* and *aflR* genes, and AFB_1_ production by *A. flavus* NRRL3357. Overall, the best reduction in *aflD *and *aflR* mRNA abundance and AFB_1 _production was at 25 nM siRNA of the concentrations tested. Statistical analysis of the effect of different concentrations of siRNA treatment on *aflD*, *aflR* gene expressions and AFB_1_ production was statistically significant (Table 2b). There was a good correlation in reduction as a result of siRNA treatment between log *aflD *and siRNA concentration (*R* = 0.86, *P* = 0.000), log AFB_1_ and siRNA concentration (*R* = 0.91, *P* = 0.000) and low correlation between log *aflR *and siRNA concentrations (*R* = 0.45, *P* = 0.06) (Table 3).

### 3.3. Treatment of *A. flavus* and *A. parasiticus* with siRNA

Table 4, Table 5 show the effect of treating three aflatoxigenic strains with the chosen concentration of the siRNA (25 nM, Nor-Ib). There was a significant effect on *aflD* (target gene), and a concomitant decrease in *aflR* mRNA abundance and AFB_1_ production by *A. flavus* EGP9 treated with siRNA when compared to the control (99.7%, 83.4%, and 89%, respectively). Treating *Aspergillus parasiticus *NRRL 13005 with siRNA revealed a reduction in *aflD* (target gene) mRNA abundance and AFG_1_ production which was statistically significant (89.4% and 77.2%, respectively). The data obtained with *Aspergillus parasiticus* SSWT 2999 after treatment with siRNA showed that there was only a significant effect in *aflD* mRNA abundance (92.3%). 

**Table 4:** Aflatoxin B_1_ (AFB_1_), Aflatoxin G_1_ (AFG_1_), *aflD* and *aflR* expression assayed by control (untreated) and Nor-1b siRNA (treated) on three aflatoxigenic strains. Each value is mean ± standard error based on three replicates.

Strains	AFB_1 _(µ/mL)		AFG_1_(µ/mL)		*AflD* Copy Numbers × 10^3^		*AflR* Copy Numbers × 10^3^	
	Un-Treated	Treated	Un-Treated	Treated	Un-Treated	Treated	Un-Treated	Treated
***A. flavus* EPG9**	0.7 ± 0.05	0.088 ± 0.003	0	0	195.6 ± 56.9	0.69 ± 0.2	41 ± 10.7	6.8 ± 0.8
***A. parasiticus*-NRRL13005**	0.15 ± 0.04	0.05 ± 0.01	2.7 ± 0.5	0.6 ± 0.2	22.9 ± 4.7	2.4 ± 1.4	0.6 ± 0.2	0.05 ± 0.007
***A. parasiticus*-SSWT2999**	1.48 ± 0.37	1.1 ± 0.4	7.8 ± 1.9	5.5 ± 3.0	499.9 ± 85.	43.6 ± 5.5	0.4 ± 0.1	0.06 ± 0.02

**Table 5:** (**a**) Analysis of Variance of the effect of siRNA (Nor-Ib) for silencing *aflD* target gene on aflatoxin B_1_, aflatoxin G_1_ expression of *aflD* and *aflR* genes of three aflatoxigenic strains (a) *A. flavus* EPG9; (**b**) *A. parasiticus*NRRL13005; and (**c**) *A. parasiticus* SSWT2999. Key: DF: Degrees of freedom, MS: mean square, P: Probability, F: F value.

	DF	MS	F	P
(**a**)				
*A. flavus* EPG9 Factor				
*aflD* copy numbers	1	5.7 × 10^10^	11.71	0.026 *
AFB_1_	1	7.4 × 10^5^	163.06	0.0002 *
(**b**)				
*A. parasiticus *NRRL13005 Factor				
*aflD* copy numbers	1	6.3 × 10^8^	17.07	0.01*
AFG1	1	6.9 × 10^6^	12.34	0.02*
(**c**)				
*A. parasiticus* SSWT2999 Factor				
*aflD* copy numbers	1	3.1 × 10^11^	28.68	0.005*
AFG1	1	8.3 × 10^6^	0.42	0.54

* Significant < 0.05 %.

## 4. Discussion

This is the first study to use RNA interference to silence one of the important structural genes in the aflatoxin biosynthesis pathway (*aflD* gene) in both *A. flavus* and *A. parasiticus* and to elucidate the function of this gene in aflatoxin production by direct delivery. Previously, it was reported that using direct delivery of dsRNAs or siRNAs could result in sequence specific suppression of this particular gene [21,22]. The application of direct delivery of synthetic siRNA, have been rarely attempted in fungi [23]. RNA interference was discovered after the injection of dsRNA into the nematode *Caenorhabditis elegans* lead to specific silencing of genes highly homologous in sequence to the delivered dsRNA [21]. Zamore *et al.* [22] reported that using the *Drosophila in vitro* system, dsRNA triggers the specific degradation of homologous RNAs only within the region of identity with dsRNA.

Our results showed that all three siRNAs designed to target *aflD *gene gave excellent levels of silencing. The transient gene silencing was observed at an early stage after 5 days of protoplast regeneration and hyphal growth, with no changes in fungal growth observed between siRNA treated and untreated samples. This suggests that protoplasts have the ability to take up siRNAs from the medium during growth. Recently, Khatri and Rajam [8] reported that germinated spores are capable of taking up siRNAs from the growth medium in the early stages of germ tube extension. 

The decrease in mRNA expression of *aflD* level caused a subsequent decrease in AFB_1_ production. Changes in AFB_1_ production in relation to mRNA level of *AflD* showed a good correlation (*r* = 0.88, *P* = 0.00001). This strongly suggest that *aflD* is absolutely essential for AFB_1_ biosynthesis and silencing of *aflD* gene expression by siRNA may result in accumulation of intermediate compounds and lead to blocking of AFB_1_ biosynthesis. In general, the aﬂatoxin gene cluster in *A. parasiticus* and *A. ﬂavus *consists of 25 genes spanning approximately 70 kb [24,25]. Aflatoxin production could be disrupted if any step in the aflatoxin biosynthetic pathway is completely blocked by a specific inhibitor. Using siRNA to target *aflD* (nor-1) gene expression that represents the early enzymatic steps in the aflatoxin biosynthetic pathway could be an appropriate target for inhibiting aflatoxin biosynthesis. Disruption or deletion of the *aflD* (nor-1) gene leads to the accumulation of norsolorinic acid and blocks the synthesis of all aflatoxins and their intermediates beyond norsolorinic acid [26]. Previously, it was reported that transformation of *A. flavus* and *A. parasiticus* with inverted repeat transgenes (IRT) containing sequence of aflatoxin-specific regulatory gene *aflR* suppressed aflatoxin production in both pathogenic fungi [27]. Also an *aflR*-specific IRT was successfully used to suppress the sterigmatocystin (ST) pathway in *A. nidulans* [28]. 

It was interesting to note that a decrease in *aflR* expression was observed following knockdown of *aflD* and changing in *AflR *mRNA levels in relation to mRNA level of *aflD* showed a good correlation (*R *= 0.82, *P* = 0.0001). One explanation for this reduction in *aflR* expression could be that there is a similarity in siRNA and the sequence of any of the global secondary metabolite regulatory machinery genes that regulate *aflR* like *LaeA*. Another explanation could be that accumulation of intermediate compounds resulting from *aflD *knockdown may have an indirect effect in suppression of *aflR* expression or any of the global secondary metabolite regulatory machinery genes. Butchko *et al.* [29] described a screen for detecting mutants defective in the sterigmatocystin (ST) gene cluster activity of *A. nidulans* by use of a genetic block early in the ST biosynthetic pathway that results in the accumulation of the first stable intermediate, norsolorinic acid. They found that three of the mutants were unable to express *aflR*, which encodes an ST zinc cluster (Zn(II)_2_ Cys_6_) transcription factor regulating ST biosynthetic gene expression. The biosynthetic and regulatory genes required for ST production in *A. nidulans* are homologous to those required for aflatoxin production in *A. flavus* and *A. parasiticus* [30,31].

The control siRNA did not lead to knockdown of *aflD* or *aflR*, suggesting that the results observed with *aflD*-specific siRNAs are not the result of a transfection artifact or an off-target effect. Our results support those obtained by Khatri and Ranjam [8]. They suggested that siRNA can cause specific silencing effects, in the polyamine biosynthetic pathway without any off-target effects. However, Jackson *et al.* [32] demonstrated that siRNAs may cross-react with targets of limited sequence similarity.

To confirm the effect of siRNA silencing, we treated three aflatoxigenic strains with siRNA. There was a significant decrease in *aflD* (targeting gene) of all three strains and an inhibition of AFB_1_ production by *A. flavus* EGP9 and AFG_1_ production by *A. parasiticus *NRRL 13005. However, there was no significant decrease in AFG_1_ by *A. parasiticus* SSWT 2999. This suggests that perhaps uptake of siRNA by *A. parasiticus* SSWT 2999 protoplasts is not as efficient as in *A. flavus *and the other strain of *A*. *parasiticus. *Thus source of the strain may influence the effectiveness of the siRNA and the threshold concentrations required may vary. Another explanation may be that the biosynthesis of aflatoxin is slightly different in *A. flavus* and *A. parasiticus*. Wilkinson *et al.* [33] reported that the regulatory mechanism or mechanisms that control aflatoxin production in *A. flavus* and *A. parasiticus* are different in response to tryptophan (Trp), where, in the presence of Trp, three aflatoxin biosynthetic pathway genes (*aflE* (norA), *aflD* (nor-1), and *aflO* (omtB)) showed a decrease in expression and AFB_1_ and AFB_2 _production for *A. flavus* while, for *A. parasiticus*, an increase in expression profile and AFB_1_ and AFG_1_ production were observed. 

## 5. Conclusions

The present study suggests that the *aflD* gene has a role in monitoring the biosynthetic direction of aflatoxin biosynthesis in *A*. *flavus* and *A*. *parasiticus.* This could thus be a good a target gene for inactivation, to develop efficient means of aflatoxin control by using RNA silencing technology. This can be applied, for example, by using mycoviruses as a candidate to mediate and propagate inactivation of the *aflD *gene.
